# Genetic Variant rs755622 Regulates Expression of the Multiple Sclerosis Severity Modifier D-Dopachrome Tautomerase in a Sex-Specific Way

**DOI:** 10.1155/2018/8285653

**Published:** 2018-07-24

**Authors:** Zhijie Han, Jiaojiao Qu, Jiehong Zhao, Xiao Zou

**Affiliations:** ^1^Innovative Drug Research and Bioinformatics Group, School of Pharmaceutical Sciences, Chongqing University, Chongqing, 401331, China; ^2^Institute of Fungus Resources, College of Life Sciences, Guizhou University, Guiyang, 550025, China; ^3^College of Pharmacy, Guiyang University of Chinese Medicine, Guian New Area, 550025, China

## Abstract

Multiple sclerosis (MS) is a sex-specific autoimmune disease involving central nervous system. Previous studies determined that macrophage migration inhibitory factor (MIF) and its homologue D-dopachrome tautomerase (DDT) sex-specifically affect MS progression. Moreover, other studies reported that rs755622 polymorphism in promoter region of* MIF* gene is associated with risk of MS and affects the promoter activity to regulate* MIF* expression in a sex-specific way. Given that* MIF* and* DDT *share a part of promoter sequence, we surmise that rs755622 can also regulate* DDT* expression in a sex-specific way. However, this has not yet been studied. Here, we used five large-scale expression quantitative trait loci (eQTLs) and two RNA-seq datasets from brain and blood to assess the potential influence of rs755622 variant on expression of* DDT* in different genders by the linear regression and differential expression analysis. The results show that the minor allele frequency of rs755622 and expression of* DDT *are significantly increased in males for MS subjects and this minor allele variant can significantly upregulate* DDT* expression for males but not females, which suggests that the regulation of* DDT* expression level by rs755622 can affect MS progression in males. These findings further support and expand conclusions of previous studies and may help to better understand the mechanisms of MS.

## 1. Introduction

Multiple sclerosis is one of the most common immune-mediated diseases involving central nervous system and presents the sex-specific rates of morbidity [1–3]. According to the report provided by* Atlas of MS* in 2013, the number of individuals affected by MS is about 2.3 million [4]. Previous Genome-wide association studies (GWAS) uncovered that about 200 variants in the human genome are associated with MS and they mainly affect immunological process related genes and lie in their regulatory regions [5, 6].

An immunoregulatory cytokine, macrophage migration inhibitory factor (MIF), plays an important role in the modulation of macrophages and microglia immune response and is associated with autoimmune and inflammation-related diseases including MS [7–11]. D-dopachrome tautomerase (DDT, also called MIF-2) is highly homologous to MIF and has a similar structure and functions as it [12–14]. Moreover, the promoter regions of* MIF* and* DDT* gene share sequences which contain some transcription factor binding sites [13, 14]. A recent study found that* DDT* is a sex-specific disease modifier for MS, and its high expression can promote MS progression in males but not females [15].

The single nucleotide polymorphism (SNP) rs755622 (G > C) lies in the promoter region of* MIF*, and previous studies showed that it is significantly associated with some immune-mediated diseases including MS [16, 17]. And then, this recent study further indicated a significant sex difference in the association between rs755622 polymorphism and MS [15]. Moreover, previous studies determined that rs755622 polymorphism affects the activity of* MIF* promoter and regulates* MIF *gene expression [18–20]. Given the relationship between* MIF *and* DDT*, we surmise that rs755622 polymorphism can also affect the expression of* DDT*. However, it is still unclear whether and how rs755622 polymorphism regulates* DDT* expression and the resulting impact on MS in different genders.

Evidence showed that regulating gene expression is an important class of the biological functions of the genetic variants [21–30], and the expression quantitative trait loci (eQTL) analysis is an effective method to discover the correlations between genetic variants and quantitative changes in gene expression [21, 22, 24, 25, 27, 31–33]. Therefore, in this study, we first selected five large-scale expression quantitative trait loci (eQTLs) datasets to assess the potential influence of rs755622 variant on expression of* DDT* in normal brain tissues and blood by a linear regression analysis. And then, we further investigated how the rs755622 polymorphism affects* DDT* expression in brain and blood of the MS patients using two RNA-seq datasets. Finally, we performed the differential expression analysis of* DDT* between genders and explored whether there is a sex-specific regulation of* DDT* expression level by the rs755622 polymorphism.

## 2. Methods

### 2.1. eQTL Analysis Using Five Large-Scale Datasets

To validate the effect of rs755622 polymorphism on* DDT* expression level, we selected five large-scale eQTL datasets which primarily consist of the European ancestry individuals without MS diagnosis [34–38]. In particular, 10 brain regions (cerebellar cortex, frontal cortex, hippocampus, medulla, occipital cortex, putamen, substantia nigra, temporal cortex, thalamus, and intralobular white matter) of 134 individuals are included in Braineac [34]; 13 brain regions (amygdala, anterior cingulate cortex, caudate, cerebellar hemisphere, cerebellum, cortex, frontal cortex, hippocampus, hypothalamus, nucleus accumbens, putamen, spinal cord, and substantia nigra) of 1,497 individuals are included in GTEx [35]; the blood of 369, 2,765, 2,116, and 5,257 individuals is included in GTEx [35], CAGE [36], BIOS QTL Browser [37], and FHS_eQTL [38], respectively. Then, we selected the rs755622 genotype and* DDT* expression data from Braineac and conducted the eQTL analysis to assess the influence of rs755622 variant on expression of* DDT* by the R package ‘Matrix eQTL', which is based on a linear regression model with the parameters, gender and age, as the covariates [39]. Finally, we analyzed the eQTL results for rs755622 (including beta and P values) from the other four datasets.

### 2.2. eQTL Analysis of MS Subjects Using the RNA-Seq Data

To further investigate the regulation of* DDT* expression by rs755622 polymorphism in MS patients, we first selected the RNA-seq data from 2 GEO datasets, GSE100297 (single-end reads produced by the Illumina HiSeq 3000) [40], and GSE66573 (paired-end reads produced by the Illumina HiSeq 2500) [41], which include the brain (optic chiasm) and blood samples of 5 and 6 MS patients, respectively. We next mapped these sequences to human reference genome (hg19) and calculated the transcript per million (TPM) values to measure* DDT* expression level in each of the MS patients using the Kallisto software, which is a quantification tool of transcript abundance based on RNA-seq data [42]. Then, these RNA-seq data were reused to detect the genotype of rs755622 polymorphism in each of the MS patients. This process includes the following: (1) the sequence reads are aligned to reference genome (hg19) using BWA software with the default parameter settings [43] and (2) SNPs are called on these aligned reads using SAMtools software with the default parameter settings (100 read depth) [44]. For the called genotype of rs755622 polymorphism, a Hardy–Weinberg Equilibrium (HWE) test based on a noncontinuity correction chi-squared method with the significance level *P* < 0.05 was performed using the R package ‘Genetics' (https://cran.r-project.org/web/packages/genetics/index.html). Finally, we used the* DDT* expression and rs755622 polymorphism genotyping data of these MS patients to conduct the eQTL analysis by the R package ‘Matrix eQTL' [39] as described in the previous step.

### 2.3. Differential Expression Analysis of DDT between Genders

According to a recent study, Benedek et al. observed that* DDT* expression level is significantly higher in brain (white matter) of male MS subjects compared with female MS subjects [15]. However, previous studies reported that the sex-specific expression has already existed in many human genes, including some MS-related genes, for the normal individuals [45, 46]. Therefore, we further explored whether the sex-specific expression of* DDT *is a general genetic model for healthy people or associated with MS. Braineac database provided the* DDT* expression data of 10 brain regions from 134 individuals free of known neurological diseases with the age and age and gender details [34]. We used these data to detect if there is significantly different expression of* DDT *between genders in the normal individuals by Student's* t*-test (the significance level was set at *P* < 0.05).

### 2.4. Effect of rs755622 Polymorphism on DDT Expression Level in Different Gender

To further explore whether the effect of rs755622 polymorphism on* DDT* expression level has a sex-specific pattern, we selected the rs755622 polymorphism genotype data of 99 male and 35 female subjects from Braineac database [34], respectively. The HWE test of rs755622 polymorphism in male and female groups was performed, respectively, using the R package ‘Genetics' as described in the previous step. And then, we used Fisher's exact test to compare the genotype frequencies of rs755622 minor allele variant (C) between genders by the R program (http://www.r-project.org/). Finally, in combination with the* DDT* expression data in the 10 brain regions, we performed the eQTL analysis to assess the influence of rs755622 variant on expression of* DDT* in males and females, respectively, by the R package ‘Matrix eQTL' [39].

## 3. Results

### 3.1. eQTL Analysis Using Five Large-Scale Datasets

Using the large-scale eQTL data from Braineac, GTEx, CAGE, BIOS QTL Browser, and FHS_eQTL, we validated the effect of rs755622 polymorphism on* DDT* expression level. Interestingly, we found a significant association between rs755622 and* DDT* expression in brain (intralobular white matter, hippocampus, cerebellum, cerebellar hemisphere, and hypothalamus) and blood when the significance level was set at *P* < 0.05. In addition, we further found that the minor allele variant (C) of rs755622 can significantly upregulate* DDT* expression (*β* > 0) in all of these tissues. More detailed information is described in Table 1.

### 3.2. eQTL Analysis of MS Subjects Using the RNA-Seq Data

According to the Ensembl database, there are 7 transcripts for the gene* DDT* (ENST00000398344.8, ENST00000350608.7, ENST00000404092.5, ENST00000430101.2, ENST00000403754.7, ENST000004 28792.1, and ENST00000444947.2) [47]. We downloaded the sequences of these transcripts as reference and calculated their TPM values in the MS patients from 2 GEO datasets using the RNA-seq data and Kallisto software. We found that the expression levels of transcripts ENST00000350608.7 and ENST00000444947.2 are generally higher comparing with the other transcripts. These results are described in Supplementary Table S1 and Table S2.

After the genotype calling of rs755622 polymorphism using these RNA-seq data, we found that there are 2 and 1 minor allele variants (C) of rs755622 in 2 MS patients from GSE100297 and GSE66573, respectively, and in other individuals no variant was detected at this polymorphism (Supplementary Table S3). And then, by setting the significance level at *P* < 0.05, we further observed that the genotype distribution of rs755622 polymorphism in the 2 groups of MS patients did not deviate from HWE (Supplementary Table S4).

Next, we performed an eQTL analysis using expression level data of the 7 transcripts for gene* DDT *in combination with genotyping data of rs755622 polymorphism. The results show that the minor allele variant (C) of rs755622 can significantly upregulate the expression of transcript ENST00000444947.2 in blood and transcript ENST00000428792.1 both in brain (optic chiasm) and blood (*P* < 0.05 and *β* > 0), which is in accordance with the eQTL results of the individuals without MS as described in the previous step. More detailed information is summarized in Table 2.

### 3.3. Differential Expression Analysis of DDT between Genders

**Figure 1:**
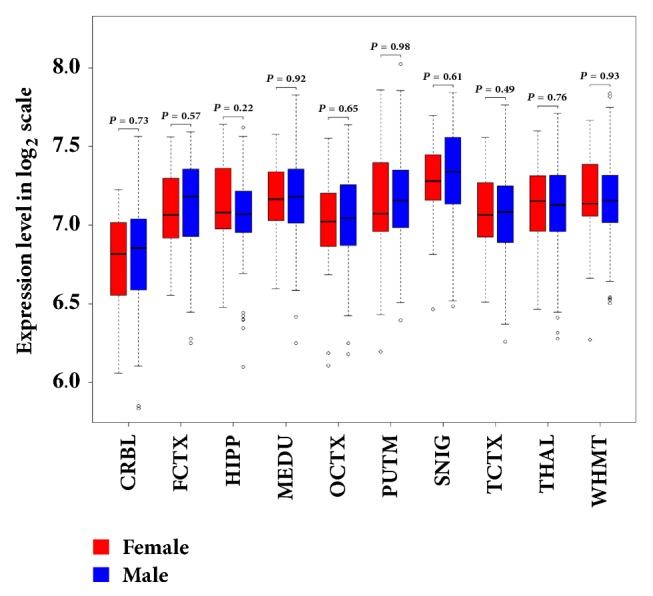
Comparison of* DDT *expression level between healthy male and female subjects in brain. Sample size in these tissues: CRBL (cerebellar cortex): 35 females and 95 males; FCTX (frontal cortex): 34 females and 93 males; HIPP (hippocampus): 30 females and 92 males; MEDU (medulla): 31 females and 88 males; OCTX (occipital cortex): 35 females and 94 males; PUTM (putamen): 33 females and 96 males; SNIG (substantia nigra): 28 females and 73 males; TCTX (temporal cortex): 33 females and 86 males; THAL (thalamus): 33 females and 91 males; WHMT (intralobular white matter): 34 females and 97 males.

To explore the association between sex-specific expression of* DDT* and MS, we compared the* DDT* expression levels between 134 males and females without known neurological diseases in 10 brain regions. The results of Student's* t*-test show that there is no significantly different expression of* DDT *between normal male and female individuals in all of the 10 brain regions (*P* < 0.05) (Figure 1). Combining Benedek et al.'s findings that* DDT* is significantly highly expressed in MS males compared to females in brain and its plasma concentration is closely related to the disease severity for MS males but not females [15], we further determined that sex-specific expression of* DDT* is associated with MS and affects the risk of MS for males.

### 3.4. Effect of rs755622 Polymorphism on DDT Expression Level in Different Gender

Because the effect of* DDT *expression on risk of MS has a sex-specific pattern, we further explored whether the regulation of rs755622 polymorphism to* DDT *expression is also different in different gender. After the HWE test, the genotype distribution of rs755622 polymorphism did not deviate from HWE (*P* < 0.05) both in male and in female groups (Supplementary Table S4). By Fisher's exact test, the frequency of the rs755622 C-containing allele (GC/CC) is no significantly different between genders for healthy subjects (22.2%  vs.  22.9%, *P* = 1), while it is significantly higher in male MS subjects compared to female MS subjects according to the results of Benedek et al.'s study (42.6%  vs.  28.2%, *P* = 4.42 × 10^−3^) [34]. More detailed information is described in Supplementary Table S5. Finally, the results of eQTL analysis show that the minor allele variant (C) of rs755622 can significantly upregulate* DDT* expression for males but not females in intralobular white matter and hippocampus (*P* < 0.05 and *β* > 0). More detailed information is summarized in Table 3.

## 4. Discussion

Both of* MIF* and* DDT*, which are highly homologous with each other, play an important role in the modulation of macrophages and microglia immune response [7, 8, 11–14]. Previous studies showed that both the* MIF *expression level and the variant of the rs755622 polymorphism in its promoter region are associated with MS progression [9, 10, 16, 17]. Moreover, other studies indicated that rs755622 polymorphism can regulate the expression of* MIF* gene by affecting the activity of its promoter [18–20]. Recently, Benedek et al. further found that* DDT* is a sex-specific disease modifier for MS [15]. Given the relationship between* MIF *and* DDT*, we surmise that rs755622 polymorphism may also sex-specifically affect the expression of* DDT *in MS.

We first assessed the influence of rs755622 variant on the expression level of* DDT* in the normal subjects using five large-scale eQTL datasets [34–38]. And then, we further used two RNA-seq datasets to genotype the rs755622 polymorphism and performed the eQTL analysis to assess this effect in MS patients. Interestingly, the results show that the minor allele variant (C) of rs755622 can significantly up-regulate* DDT* expression level (*P* < 0.05 and *β* > 0) in brain tissues (intralobular white matter, hippocampus, cerebellum, cerebellar hemisphere, hypothalamus, and optic chiasm) and blood both for normal and MS subjects.

The differential expression analysis shows that there is no significant difference in the expression level of* DDT *between the healthy male and female subjects. In combination with the results of Benedek et al.'s study [15], we further determined that the* DDT* is highly expressed in MS brain tissues and promotes MS progression for males but not females.

Finally, by the eQTL analysis on male and female subjects using Braineac data [34], respectively, we found that the minor allele variant (C) of rs755622 can significantly upregulate* DDT* expression for males but not females. In addition, we also found that the distribution of the rs755622 polymorphism genotype is not significantly different between genders for healthy subjects, but the frequency of its minor allele variant (C) is significantly higher in male MS subjects according to Benedek et al.'s study [15]. Therefore, we think that both the rs755622 polymorphism sex-specific regulation and its different genotype distribution between genders in MS contribute to the high expression level of* DDT* in MS males.

In summary, expression level of* DDT* is significantly high in male MS subjects comparing with females and closely related to the disease severity of MS in males [15]. There is a MS-related SNP rs755622 in the promoter region of* MIF *which is highly homologous to* DDT*, and the frequency of its minor allele variant (C) is significantly increased and associated with the high expression level of* DDT* in male MS subjects. These results indicate that the regulation of* DDT* expression level by the rs755622 polymorphism can affect MS progression in males, which further supports and expands the findings in previous studies [12–17] and may help to better understand the mechanisms of MS.

## Figures and Tables

**Table 1:** Polymorphism rs755622 C allele upregulates *DDT* expression in brain and blood for subjects without MS diagnosis.

Gene	SNP	Effect allele	P value	Effect size (*β*)	Tissues	Sample size	Dataset
*DDT*	rs755622	C	1.47E-02*∗*	0.1282	Brain intralobular white matter	134	Braineac
*DDT*	rs755622	C	3.97E-02*∗*	0.1013	Brain hippocampus	134	Braineac
*DDT*	rs755622	C	1.66E-01	-0.0874	Brain cerebellar cortex	134	Braineac
*DDT*	rs755622	C	5.84E-01	0.0284	Brain frontal cortex	134	Braineac
*DDT*	rs755622	C	3.61E-01	-0.0436	Brain medulla	134	Braineac
*DDT*	rs755622	C	4.27E-01	-0.0432	Brain occipital cortex	134	Braineac
*DDT*	rs755622	C	9.63E-01	-0.0027	Brain putamen	134	Braineac
*DDT*	rs755622	C	2.09E-01	-0.0630	Brain substantia nigra	134	Braineac
*DDT*	rs755622	C	6.74E-01	0.0219	Brain temporal cortex	134	Braineac
*DDT*	rs755622	C	3.87E-01	-0.0462	Brain thalamus	134	Braineac
*DDT*	rs755622	C	5.09E-01	-0.0743	Brain amygdala	88	GTEx
*DDT*	rs755622	C	3.28E-01	-0.1050	Brain anterior cingulate cortex	109	GTEx
*DDT*	rs755622	C	8.55E-02	0.1732	Brain caudate	144	GTEx
*DDT*	rs755622	C	3.16E-01	0.1241	Brain cortex	136	GTEx
*DDT*	rs755622	C	1.61E-01	0.1353	Brain frontal cortex	118	GTEx
*DDT*	rs755622	C	7.21E-01	-0.0383	Brain hippocampus	111	GTEx
*DDT*	rs755622	C	2.84E-01	0.1136	Brain nucleus accumbens	130	GTEx
*DDT*	rs755622	C	7.33E-01	-0.0417	Brain putamen	111	GTEx
*DDT*	rs755622	C	1.24E-01	0.2706	Brain spinal cord	83	GTEx
*DDT*	rs755622	C	7.01E-01	-0.0553	Brain substantia nigra	80	GTEx
*DDT*	rs755622	C	1.21E-04*∗*	0.5072	Brain cerebellum	154	GTEx
*DDT*	rs755622	C	1.21E-03*∗*	0.3480	Brain cerebellar hemisphere	125	GTEx
*DDT*	rs755622	C	2.91E-02*∗*	0.2388	Brain hypothalamus	108	GTEx
*DDT*	rs755622	C	4.62E-06*∗*	0.2260	Blood	369	GTEx
*DDT*	rs755622	C	3.70E-35*∗*	0.4214	Blood	2,765	CAGE
*DDT*	rs755622	C	4.31E-28*∗*	> 0 (z =10.989)	Blood	2,116	BIOS QTL Browser
*DDT*	rs755622	C	5.21E-16*∗*	0.0427	Blood	5,257	FHS_eQTL

The rs755622 position (hg19), 22_24236392_G_C_b37 (G > C); the threshold of significant association is 0.05; *β* > 0 and *β* < 0 means that this effect allele upregulates and downregulates gene expression, respectively; *β* = z × SE  (standard  errors); the asterisk (*∗*) means a significant association.

**Table 2:** Polymorphism rs755622 C allele upregulates *DDT* expression in brain and blood for MS patients.

Transcript ID	Gene	SNP	Effect allele	P value	Effect size (*β*)	Tissues	GEO dataset
ENST00000428792.1	*DDT*	rs755622	C	2.23E-308	6.72E+03	Brain optic chiasm	GSE100297
ENST00000428792.1	*DDT*	rs755622	C	2.91E-15	3.60E+01	Blood	GSE66573
ENST00000444947.2	*DDT*	rs755622	C	1.62E-02	1.23E+05	Blood	GSE66573

The rs755622 position (hg19), 22_24236392_G_C_b37 (G > C); the threshold of significant association is 0.05; *β* > 0 and *β* < 0 means that this effect allele upregulates and downregulates gene expression, respectively.

**Table 3:** Regulation of rs755622 polymorphism to *DDT* expression level in different gender.

Gender	Gene	SNP	Effect allele	P value	Effect size (*β*)	Tissues	Sample size
Female+Male	*DDT*	rs755622	C	**2.80E-02**	1.29E-01	Brain hippocampus	122
Female	*DDT*	rs755622	C	1.05E-01	1.96E-01	Brain hippocampus	30
Male	*DDT*	rs755622	C	**4.41E-02**	1.40E-01	Brain hippocampus	92
Female+Male	*DDT*	rs755622	C	**2.26E-02**	1.24E-01	Brain intralobular white matter	131
Female	*DDT*	rs755622	C	3.14E-01	1.28E-01	Brain intralobular white matter	34
Male	*DDT*	rs755622	C	**2.03E-02**	1.46E-01	Brain intralobular white matter	97

Significant associations (P < 0.05) marked in bold. The rs755622 position (hg19), 22_24236392_G_C_b37 (G > C); the threshold of significant association is 0.05; *β* > 0 and *β* < 0 means that this effect allele up-regulates and down-regulates gene expression, respectively.

## Data Availability

The data used to support the findings of this study are available from the corresponding author upon request.

## Supplementary Materials

Supplementary Materials Table S1: the expression levels of 7 transcripts for the gene DDT in MS patients from GSE100297. Table S2: the expression levels of 7 transcripts for the gene DDT in MS patients from GSE66573. Table S3: the genotype of rs755622 polymorphism in MS patients from 2 GEO dataset. Table S4: the results of HWE test for genotype distribution of rs755622 polymorphism in different groups. Table S5: the genotype distribution of rs755622 polymorphism between gender in MS and healthy subjects.
