# Supplementary material for: Genetic Variant rs755622 Regulates Expression of the Multiple Sclerosis Severity Modifier D-Dopachrome Tautomerase in a Sex-Specific Way
Source: Biomed Res Int. 2018 Jul 24;2018:8285653. doi: 10.1155/2018/8285653 (PMC6081589; doi:10.1155/2018/8285653)
Supplement: Supplementary Materials — Table S1: the expression levels of 7 transcripts for the gene DDT in MS patients from GSE100297. Table S2: the expression levels of 7 transcripts for the gene DDT in MS patients from GSE66573. Table S3: the genotype of rs755622 polymorphism in MS patients from 2 GEO dataset. Table S4: the results of HWE test for genotype distribution of rs755622 polymorphism in different groups. Table S5: the genotype distribution of rs755622 polymorphism between gender in MS and healthy subjects. [file 8285653.f1.docx]

Supplementary Data

**Genetic variant rs755622 regulates expression of the multiple sclerosis severity modifier D-dopachrome tautomerase in a sex-specific way**

Zhijie Han**^1^**, Jiaojiao Qu**^2^**, Jiehong Zhao**^3^**, Xiao Zou**^2*^**

1 Innovative Drug Research and Bioinformatics Group, School of Pharmaceutical Sciences, Chongqing University, Chongqing, 401331, China

2 Institute of Fungus Resources, College of Life Sciences, Guizhou University, Guiyang, 550025, China.

3 College of Pharmacy, Guiyang University of Chinese Medicine, Guian new area, 550025, China.

* Corresponding Author: Xiao Zou ([xzou@gzu.edu.cn](mailto:xzou@gzu.edu.cn))

**Table S1** The expression levels of 7 transcripts for the gene *DDT* in MS patients from GSE100297

| Gene | Transcripts | Length (bp) | Expression levels (TPM) | | | | |
| --- | --- | --- | --- | --- | --- | --- | --- |
|  |  |  | SRR5724993 | SRR5724994 | SRR5724995 | SRR5724996 | SRR5724997 |
| *DDT* | ENST00000398344.8 | 713 | 9.76E+00 | 1.27E+01 | 5.84E+00 | 5.98E+00 | 9.67E+00 |
| *DDT* | ENST00000350608.7 | 671 | 9.30E+05 | 8.74E+05 | 8.85E+05 | 8.47E+05 | 8.35E+05 |
| *DDT* | ENST00000404092.5 | 805 | 3.24E+04 | 2.26E+04 | 3.71E+04 | 8.13E+04 | 4.71E+04 |
| *DDT* | ENST00000430101.2 | 573 | 0.00E+00 | 5.69E+03 | 0.00E+00 | 0.00E+00 | 0.00E+00 |
| *DDT* | ENST00000403754.7 | 569 | 0.00E+00 | 1.82E+04 | 0.00E+00 | 1.31E+04 | 1.24E+04 |
| *DDT* | ENST00000428792.1 | 333 | 0.00E+00 | 0.00E+00 | 0.00E+00 | 0.00E+00 | 1.34E+04 |
| *DDT* | ENST00000444947.2 | 1048 | 3.77E+04 | 7.99E+04 | 7.75E+04 | 5.87E+04 | 9.17E+04 |

TPM: the transcript per million values.

**Table S2** The expression levels of 7 transcripts for the gene *DDT* in MS patients from GSE66573

| Gene | Transcripts | Length (bp) | Expression levels (TPM) | | | | | |
| --- | --- | --- | --- | --- | --- | --- | --- | --- |
|  |  |  | SRR1839792 | SRR1839793 | SRR1839795 | SRR1839796 | SRR1839797 | SRR1839798 |
| *DDT* | ENST00000398344.8 | 713 | 1.52E+05 | 1.75E+05 | 1.63E+01 | 1.47E+05 | 3.57E+04 | 1.58E+01 |
| *DDT* | ENST00000350608.7 | 671 | 7.74E+05 | 7.72E+05 | 9.03E+05 | 7.37E+05 | 8.67E+05 | 9.37E+05 |
| *DDT* | ENST00000404092.5 | 805 | 1.47E+00 | 5.28E+00 | 0.00E+00 | 6.34E-02 | 2.29E-02 | 0.00E+00 |
| *DDT* | ENST00000430101.2 | 573 | 0.00E+00 | 1.30E+04 | 0.00E+00 | 5.90E+03 | 3.67E+00 | 0.00E+00 |
| *DDT* | ENST00000403754.7 | 569 | 2.95E+04 | 3.64E+00 | 0.00E+00 | 1.76E-05 | 7.06E+00 | 0.00E+00 |
| *DDT* | ENST00000428792.1 | 333 | 0.00E+00 | 0.00E+00 | 0.00E+00 | 3.60E+01 | 1.09E-02 | 0.00E+00 |
| *DDT* | ENST00000444947.2 | 1048 | 4.38E+04 | 4.01E+04 | 9.74E+04 | 1.91E+05 | 9.76E+04 | 6.27E+04 |

TPM: the transcript per million values.

**Table S3** The genotype of rs755622 polymorphism in MS patients from 2 GEO dataset

| SNP | Position (hg19) | Minor allele | Genotype | Individuals | GEO dataset |
| --- | --- | --- | --- | --- | --- |
| rs755600 | 8247877 | C | G/G | SRR5724993 | GSE100297 |
| rs755600 | 8247877 | C | G/G | SRR5724994 | GSE100297 |
| rs755600 | 8247877 | C | G/G | SRR5724995 | GSE100297 |
| rs755600 | 8247877 | C | G/G | SRR5724996 | GSE100297 |
| rs755600 | 8247877 | C | C/C | SRR5724997 | GSE100297 |
| rs755600 | 8247877 | C | G/G | SRR1839792 | GSE66573 |
| rs755600 | 8247877 | C | G/G | SRR1839793 | GSE66573 |
| rs755600 | 8247877 | C | G/G | SRR1839795 | GSE66573 |
| rs755600 | 8247877 | C | G/C | SRR1839796 | GSE66573 |
| rs755600 | 8247877 | C | G/G | SRR1839797 | GSE66573 |
| rs755600 | 8247877 | C | G/G | SRR1839798 | GSE66573 |

**Table S4** The results of HWE test for genotype distribution of rs755622 polymorphism in different groups

| Dataset | Gender | GG | GC | CC | MAF | X-squared | P value |
| --- | --- | --- | --- | --- | --- | --- | --- |
| Braineac | mixed | 104 | 28 | 2 | 0.136 | 0.005 | 1.00E+00 |
| Braineac | female | 27 | 8 | 0 | 0.129 | 0.583 | 1.00E+00 |
| Braineac | male | 77 | 20 | 2 | 0.138 | 0.265 | 6.33E-01 |
| GSE100297 | mixed | 4 | 0 | 1 | 0.250 | 5.000 | 2.02E-01 |
| GSE66573 | mixed | 5 | 1 | 0 | 0.091 | 0.050 | 1.00E+00 |

The threshold of significant deviation from HWE is $P<0.05$.

**Table S5** the genotype distribution of rs755622 polymorphism between gender in MS and healthy subjects

| SNP | Genotype | Female, n (%) | Male, n (%) | P value | Phenotype | Dataset |
| --- | --- | --- | --- | --- | --- | --- |
| rs755622 | GG | 27 (77.1) | 77 (77.8) | 1.00E+00 | healthy | Braineac |
| rs755622 | C containing | 8 (22.9) | 22 (22.2) |  | healthy | Braineac |
| rs755622 | GG | 79 (71.8) | 351 (57.4) | 4.42E-03 | MS | PMID: 28923927 |
| rs755622 | C containing | 31 (28.2) | 260 (42.6) |  | MS | PMID: 28923927 |

The *P* values are calculated by Fisher’s exact test.
